# Fast adiabatic quantum state transfer and entanglement generation between two atoms via dressed states

**DOI:** 10.1038/srep46255

**Published:** 2017-04-11

**Authors:** Jin-Lei Wu, Xin Ji, Shou Zhang

**Affiliations:** 1 Department of Physics, College of Science, Yanbian University, Yanji, Jilin 133002, People’s Republic of China

## Abstract

We propose a dressed-state scheme to achieve shortcuts to adiabaticity in atom-cavity quantum electrodynamics for speeding up adiabatic two-atom quantum state transfer and maximum entanglement generation. Compared with stimulated Raman adiabatic passage, the dressed-state scheme greatly shortens the operation time in a non-adiabatic way. By means of some numerical simulations, we determine the parameters which can guarantee the feasibility and efficiency both in theory and experiment. Besides, numerical simulations also show the scheme is robust against the variations in the parameters, atomic spontaneous emissions and the photon leakages from the cavity.

Quantum state transfer and entanglement generation between different systems with time-dependent interacting fields have become more and more important for the further development of quantum information processing^1^,^2^,^3^. In order to achieve the high-fidelity quantum state transfer and entanglement generation, adiabatic evolution which corresponds to a state transfer along the adiabatic eigenstates is an excellent candidate method^4^,^5^. The most widely used approach of adiabatic evolution is stimulated Raman adiabatic passage (STIRAP) because of its great robustness against pulse area and timing errors as well as the restraint on lossy intermediate states.

However, STIRAP schemes usually require a relatively long interaction time, and thus the adiabatic evolution may suffer from dissipation and noise during the process of quantum state transfer. Therefore, it is greatly necessary to speed up the process of adiabatic evolution and lots of theoretical schemes have been brought forward^6^,^7^,^8^,^9^,^10^,^11^,^12^,^13^,^14^,^15^,^16^,^17^,^18^. Besides, some experimental realizations have been completed^19^,^20^,^21^,^22^,^23^. There are two techniques, transitionless quantum driving and Lewis-Riesenfeld invariants, widely applied to speed up adiabatic quantum state transfer and entanglement generation^24^,^25^,^26^,^27^,^28^,^29^,^30^. Although, in theory, the high-fidelity adiabatic quantum state transfer and entanglement generation can be achieved by transitionless quantum driving and Lewis-Riesenfeld invariants, it is hardly possible in practice due to the major flaws of the two techniques. On one hand, a transitionless-based direct coupling between the initial state and the target state is too hard to be obtained experimentally^31^,^32^,^33^. On the other hand, in some schemes^34^,^35^,^36^,^37^,^38^, invariants-based driving pulses are not smoothly turned on or off and thus lead to severe impediments in experiment.

A short time before, Baksic *et al*. proposed a new method to speed up adiabatic quantum state transfer by using dressed states^39^. In ref. 39, the dressed states are skillfully defined to incorporate the nonadiabatic processes. Kang *et al*. used the dressed-state method to implement the entanglement generation in a solid quantum system^40^. In this work, we apply the dressed-state method to quantum state transfer and entanglement generation between two Λ-type atoms trapped in an optical cavity. With the assist of quantum Zeno dynamics^41^,^42^, the original system Hamiltonian is simplified and viewed as a three-level system. With the addition of a suitable correction Hamiltonian to the original Hamiltonian and the ingenious unitary transformation, we construct a new diagonal Hamiltonian in the time-independent dressed-state frame. Then the adiabatic two-atom quantum state transfer and entanglement generation are speeded up in a non-adiabatic way.

## Adiabatic quantum state transfer and entanglement generation between two atoms

**Figure 1:**
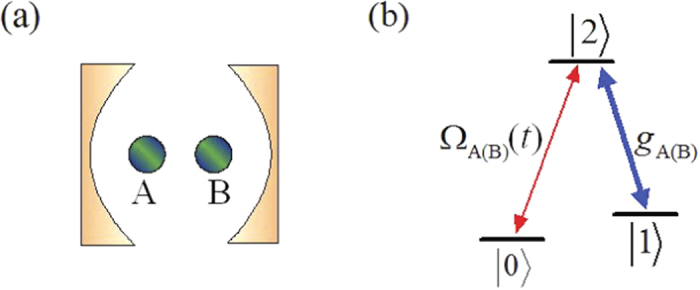
(**a**) The diagrammatic sketch of cavity-atom combined system. (**b**) Atomic level configuration.

The schematic setup for quantum state transfer and entanglement generation between two atoms is shown in Fig. 1. Two Λ-type atoms A and B are trapped in a single-mode optical cavity. Each atom has an excited state |2〉 and two ground states |0〉 and |1〉. The atomic transition |2〉_A(B)_ ↔ |1〉_A(B)_ is resonantly coupled to the mode of the cavity with corresponding coupling constant *g*_A(B)_, and the transition |2〉_A(B)_ ↔ |0〉_A(B)_ is resonantly driven by a classical field with the time-dependent Rabi frequency Ω_A(B)_(*t*). Then the atom-cavity system can be dominated by the interaction Hamiltonian (setting *ħ* = 1)


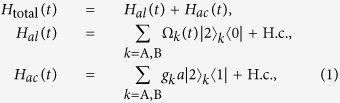


where *H*_*al*_(*t*) (*H*_*ac*_(*t*)) is the interaction between the atoms and the classical laser fields (the mode of the cavity), and *a* is the annihilation operator of the cavity mode. For simplicity, we assume *g*_A_ = *g*_B_ = *g*. Then suppose that the total system is initially in the state |*ϕ*_1_〉 = |0〉_A_|1〉_B_|0〉_c_ denoting atom A, atom B and the cavity mode in the state |0〉_A_, state |1〉_B_ and vacuum state, respectively. Thus dominated by the Hamiltonian (1), the whole system evolves in the Hilbert space spanned by


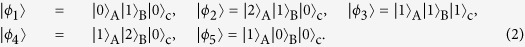


Obviously, the system is initially in the dark state of *H*_*ac*_(*t*), i.e., *H*_*ac*_(*t*)|*ϕ*_1_〉 = 0. Then choosing the quantum Zeno limit condition 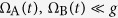, the whole system can approximatively evolve in an invariant Zeno subspace consisting of dark states of *H*_*ac*_(*t*)^43^,^44^


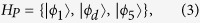


corresponding to the projections


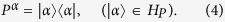


Here,


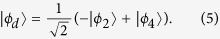


Therefore, the system Hamiltonian can be rewritten as the following form^45^


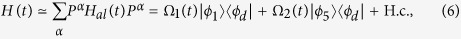


in which 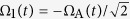 and 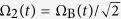. Then the pulses are parameterized by the frequency Ω(*t*) and the angle *θ*(*t*)


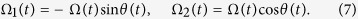


with 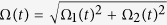 and *θ*(*t*) = *arctan*(Ω_1_(*t*)/Ω_2_(*t*)), and we can easily obtain the time-dependent eigenstates of *H*(*t*)


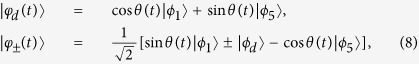


with the eigenvalues *E*_*d*_ = 0 and *E*_±_  = ±Ω(*t*), respectively.

Next we move the Hamiltonian (6) to the time-independent adiabatic frame defined by the unitary operator *U*(*t*) = ∑_*j*=*d*,±_|*φ*_*j*_〉〈*φ*_*j*_(*t*)|, and the Hamiltonian (6) becomes


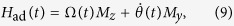


with *M*_*z*_ = |*φ*_+_〉〈*φ*_+_| − |*φ*_−_〉〈*φ*_−_| and 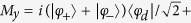 H.c. The second term of the Hamiltonian (9) corresponds to the nonadiabatic couplings which may lead to an imperfect state transfer. In order to correct the nonadiabatic errors, we look for a correction Hamiltonian *H*_*c*_(*t*) such that the modified Hamiltonian *H*_mod_(*t*) = *H*(*t*) + *H*_*c*_(*t*) governs a perfect state transfer. Here we choose the general form of *H*_*c*_(*t*)


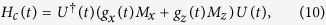


where we introduce 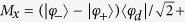
H.c., and *g*_*x*_(*t*) and *g*_*z*_(*t*) are two parameters determined later. Then the Hamiltonian (6) becomes


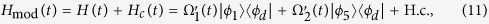


with the modified pulses


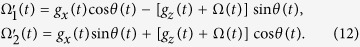


We define a new basis of dressed states 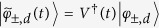. In this scheme, we choose


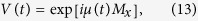


with an Euler angle *μ*(*t*). By moving the modified Hamiltonian (11) to the dressed-state frame defined by *V*(*t*), we obtain a new Hamiltonian


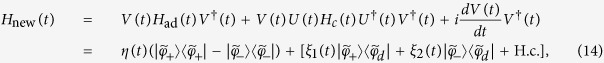


with 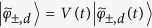 and the time-dependent parameters 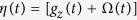 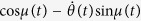, 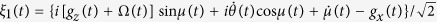 and 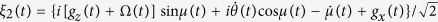. When the parameters are chosen as


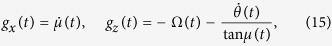


the second term of the Hamiltonian (14) is removed. There will be no transition between 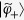, 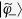 and 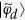. Furthermore, in the original frame, the transitions between 
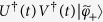
, 
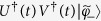
 and 
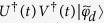
 are canceled.

Next, in the original frame, we define


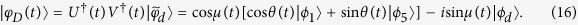


Obviously, |*φ*_*D*_(*t*)〉 can serve as a medium state which operates a two-atom quantum state transfer |*ϕ*_1_〉 → |*ϕ*_5_〉 by setting *θ*(*t*_*i*_) = 0, *θ*(*t*_*f*_) = *π*/2 and *μ*(*t*_*i*_) = *μ*(*t*_*f*_) = 0, where *t*_*i*(*f*)_ is the initial (final) time. Analogously, a maximum two-atom entangled state 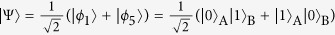 can be generated by setting *θ*(*t*_*i*_) = 0, *θ*(*t*_*f*_) = *π*/4 and *μ*(*t*_*i*_) = *μ*(*t*_*f*_) = 0.

Based on the process above, we have used the dressed-state method to achieve shortcuts to adiabaticity in a non-adiabatic way and implement the fast two-atom quantum state transfer and maximum entanglement generation. The evolution process is not necessarily slow and there is no direct coupling between the initial state and the target state, as long as a set of suitable dressed states is chosen.

## Numerical simulations

### Selections of parameters

First of all, we give numerical simulations to select appropriate parameters for insuring the experimental and theoretical feasibility. The original pulses Ω_1_(*t*) and Ω_2_(*t*) can be chosen as the Gaussian pulses^1^,^4^


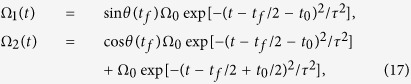


and the corresponding 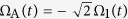 and 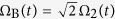, the original driving pulses of the total system, can be obtained. In the scheme, we choose *t*_0_ = 3*t*_*f*_/40 and *τ* = 0.1*t*_*f*_. Besides, *θ*(*t*_*f*_) = *π*/2 and *θ*(*t*_*f*_) = *π*/4 are for the quantum state transfer and the entanglement generation, respectively. The Euler angle *μ*(*t*) is defined by


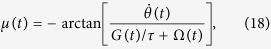


where *G*(*t*) = sech(*t*/*τ*) is chosen to regularize *μ*(*t*) such that it can meet the condition *μ*(*t*_*i*_) = *μ*(*t*_*f*_) = 0 and make 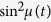, the population of |*ϕ*_*d*_〉 (see Equation (16)), as small as possible. Then based on the relevant parameters above, the modified pulses 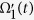 and 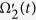 can be determined by Eq. (12). Also, the corresponding modified driving pulses of the total system 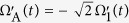 and 
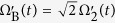
 are obtained.

**Figure 2:**
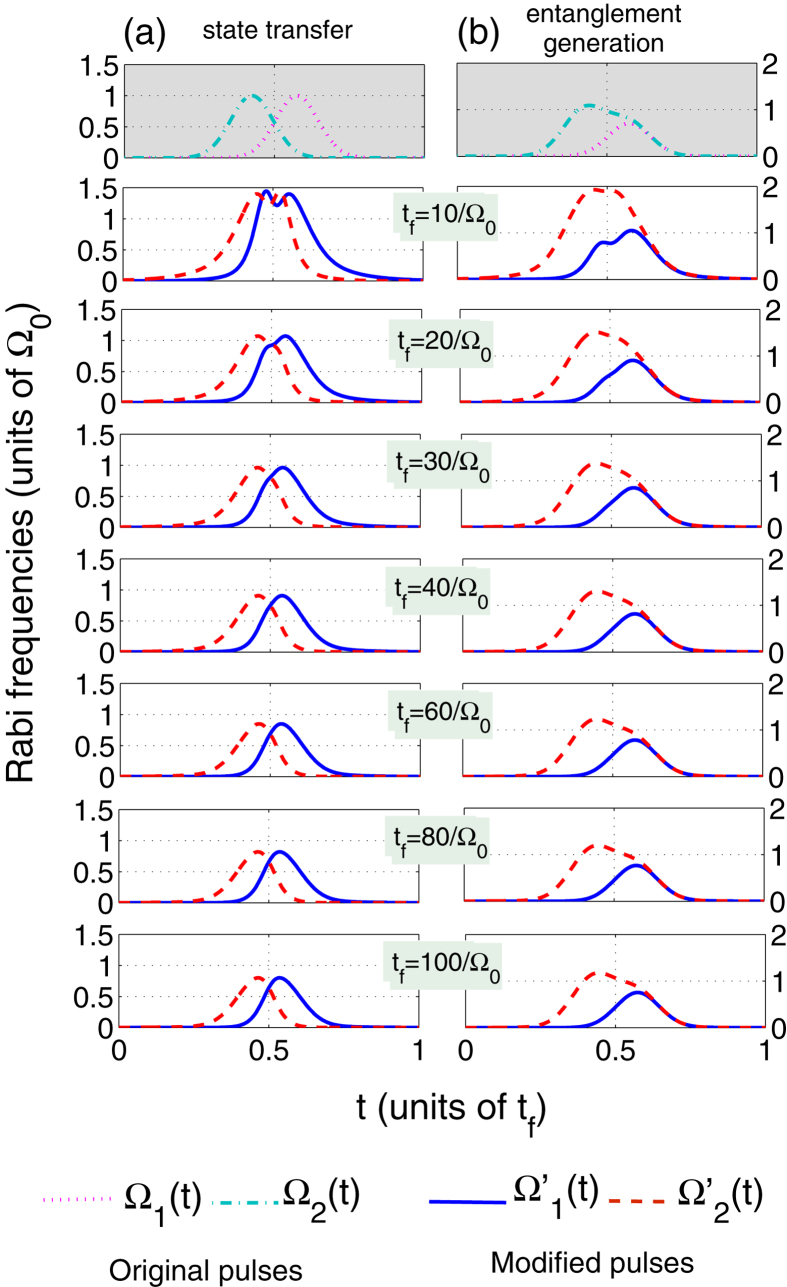
The shapes of the modified pulses 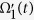 and 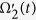 with several different values of the operation time *t*_*f*_. The parameters used here are *t*_0_ = 3*t*_*f*_/40, *τ* = 0.1*t*_*f*_, *θ*(*t*_*f*_) = *π*/2 for the quantum state transfer or *θ*(*t*_*f*_) = *π*/4 for the entanglement generation.

**Figure 3:**
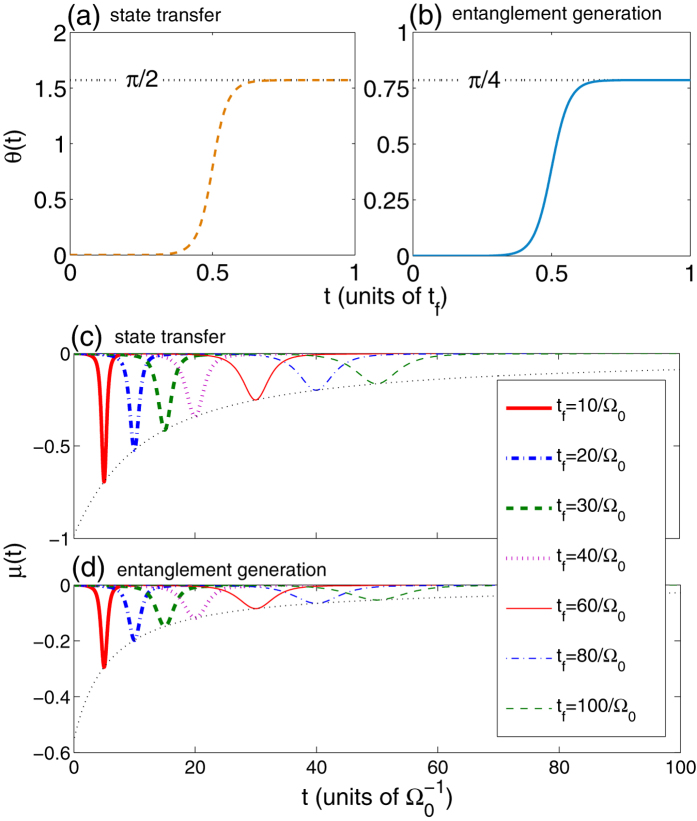
(**a**,**b**) Time dependence of *θ*(*t*) with an arbitrary *t*_*f*_; (**c**,**d**) time dependence of *μ*(*t*) with some different *t*_*f*_. The parameters used here are the same as in Fig. 2.

In order to choose a small and feasible value of the operation time *t*_*f*_, in Fig. 2, we plot the shapes of the modified pulses 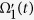 and 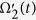 with different values of *t*_*f*_. From Fig. 2, we can clearly find that, both for the quantum state transfer and the entanglement generation, the longer the operation time *t*_*f*_ is, the more similar the shapes of the modified pulses are to those of the original pulses. In other words, the modified pulses’ experimental feasibility increases as the operation time *t*_*f*_ increases, which implies the operation time we choose can not be too short. Then, taking the boundary conditions about *θ*(*t*) into account, we plot the time dependence of *θ*(*t*) in Fig. 3(a,b) for the quantum state transfer and the entanglement generation, respectively, with an arbitrary *t*_*f*_. Without a doubt, Fig. 3(a,b) show that the boundary conditions with respect to *θ*(*t*) can be satisfied perfectly, and the time dependence of *θ*(*t*) is independent of *t*_*f*_. For the boundary condition *μ*(*t*_*i*_) = *μ*(*t*_*f*_) = 0, we show it in Fig. 3(c,d) by plotting the time dependence of *μ*(*t*) with several different values of *t*_*f*_. Apparently, *μ*(*t*_*i*_) = *μ*(*t*_*f*_) = 0 is always satisfied well with an arbitrary *t*_*f*_. The maximum values of |*μ*(*t*)|, however, always decrease with the increase of *t*_*f*_. Therefore, for |*ϕ*_*d*_〉 population sin^2^*μ*(*t*) being small enough, the operation time we choose can not be too short.

**Figure 4:**
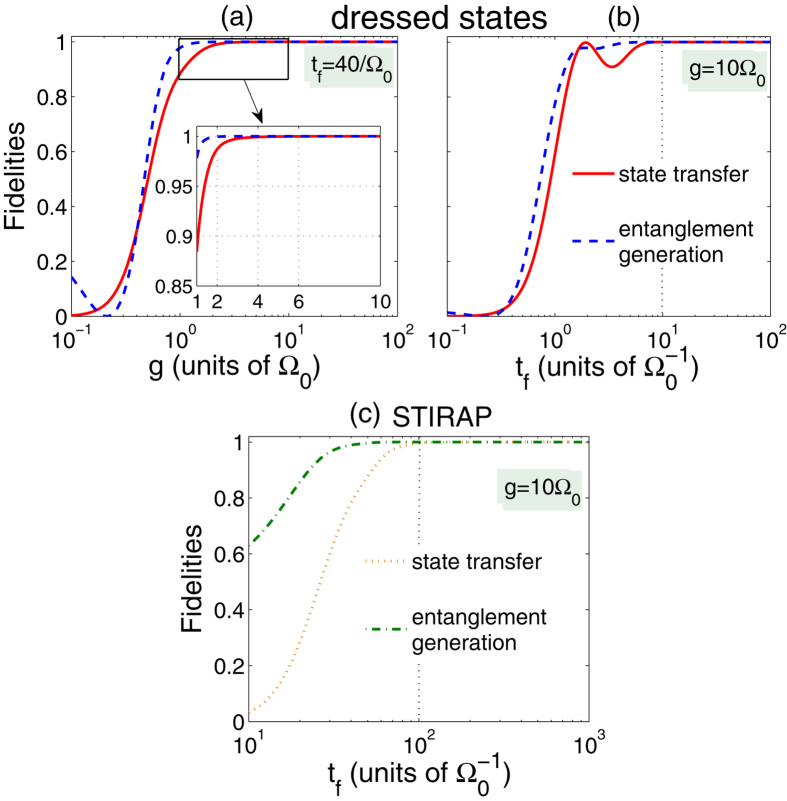
(**a**) Fidelities as functions of *g* with *t*_*f*_ = 40/Ω_0_ in the dressed-state scheme; (**b**) fidelities as functions of *t*_*f*_ with *g* = 10 Ω_0_ in the dressed-state scheme; (**c**) fidelities as functions of *t*_*f*_ with *g* = 10 Ω_0_ in the STIRAP scheme. Other parameters used here are the same as in Fig. 2.

For the high experimental feasibility of the scheme and a relatively small occupancy of |*ϕ*_*d*_〉, we preselect *t*_*f*_ = 40/Ω_0_. Then with *t*_*f*_ = 40/Ω_0_, in Fig. 4(a), we plot the fidelities of the dressed-state scheme as functions of the atom-cavity coupling strength *g*, where the fidelities are defined by *F* = |〈*ϕ*_*ideal*_|*ϕ*(*t*)〉|^2^ with |*ϕ*_*ideal*_〉 = |*ϕ*_5_〉 for the state transfer or 
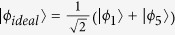
 for the entanglement generation. |*ϕ*(*t*)〉 is the state of the whole system. Because of the Zeno limit condition 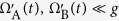, the fidelities increase with the increase of *g*/Ω_0_. However, as shown in Fig. 4(a), even when *g* = 4 Ω_0_ which does not strictly meet the Zeno limit condition, the fidelities are almost unit both for the quantum state transfer and the entanglement generation. Here we preselect *g* = 10 Ω_0_ to guarantee the scheme’s robustness. For checking the feasibility of the preselection *t*_*f*_ = 40/Ω_0_, we plot the fidelities as functions with respect to *t*_*f*_ in Fig. 4(b) with *g* = 10 Ω_0_, and Fig. 4(b) indicates that the preselection *t*_*f*_ = 40/Ω_0_ is feasible and robust both for the quantum state transfer and the entanglement generation. Next, in Fig. 4(c), we plot the fidelities versus *t*_*f*_ in the STIRAP scheme. Through the comparison between Fig. 4(b,c), we know that the operation time in the dressed-state scheme is just around 1/10 of that in the STIRAP scheme when the fidelities reach near 1, which indicates that the dressed-state scheme can greatly speed up the adiabatic two-atom quantum state transfer and entanglement generation.

### Discussion of effectiveness

**Figure 5:**
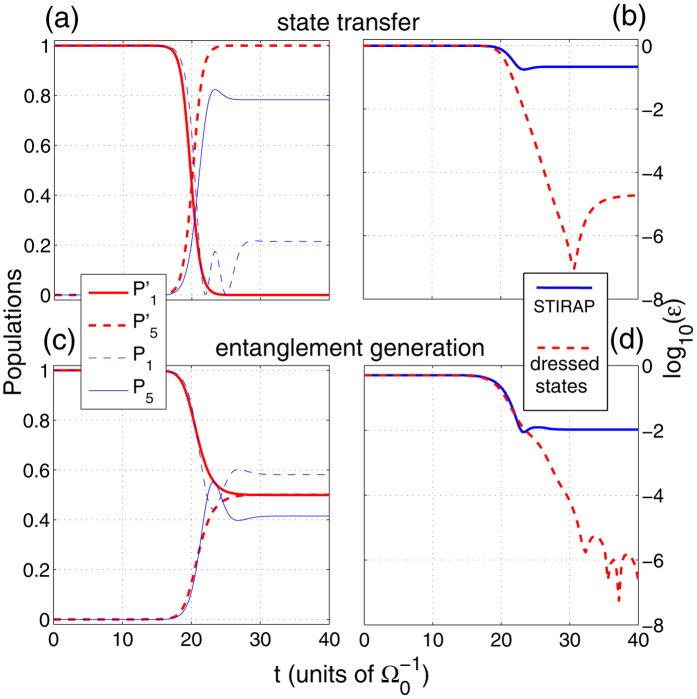
(**a**,**c**): Time dependence of the populations of the states |*ϕ*_1_〉 and |*ϕ*_5_〉 in the dressed-state scheme (thick red lines) and the STIRAP scheme (thin blue lines); (**b**,**d**): residual errors of the states in the dressed-state scheme (red dashed lines) and the STIRAP scheme (blue solid lines). *g* = 10 Ω_0_, *t*_*f*_ = 40/Ω_0_ and other parameters used here are the same as in Fig. 2.

In this subsection, in order to show the effectiveness of the dressed-state scheme, in Fig. 5, we show the time dependence of the populations of the states |*ϕ*_1_〉 and |*ϕ*_5_〉 and the residual errors *ε*(*t*) = 1 − |〈*ϕ*_*ideal*_|*ϕ*(*t*)〉|^2^ of the quantum state transfer and the entanglement generation, respectively. As shown in Fig. 5(a,c), the dressed-state scheme achieves the perfect desired population transfer both for the quantum state transfer and the entanglement generation, but the STIRAP scheme can not perfectly achieve the quantum state transfer or the entanglement generation. Correspondingly, in Fig. 5(b,d), the dressed-state scheme leads to a reduction of the residual errors by over four orders of magnitude at the final time both for the state transfer and the entanglement generation. Therefore, there is no doubt that the dressed-state scheme is highly feasible and effective even within a very short operation time.

### Discussion of robustness

In the above discussion, we think the operations and the whole system perfect and absolutely isolated from the environment. Therefore, it is necessary to give the discussions about the robustness of the scheme against the variations in the parameters and decoherence induced by the atomic spontaneous emissions and photon leakages from the cavity.

**Figure 6:**
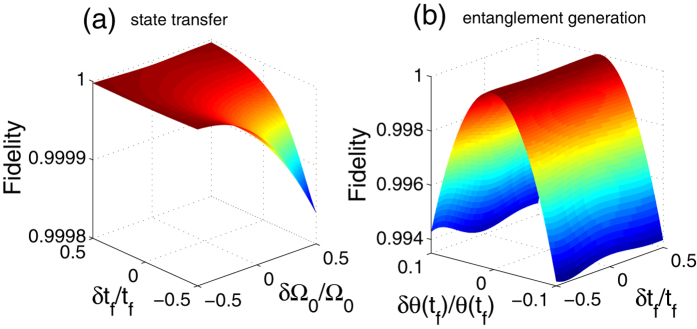
(**a**) The fidelity versus the variations in the pulses time *t*_*f*_ and amplitude Ω_0_ for the state transfer; (**b**) the fidelity versus the variations in *t*_*f*_ and *θ*(*t*_*f*_) for the entanglement generation. The parameters used here are same as in Fig. 5.

We first consider the robustness of the scheme against variations in the parameters of the pulse sequences by plotting the fidelity versus the variations in the pulses time *t*_*f*_ and amplitude Ω_0_ in Fig. 6(a) for the two-atom state transfer based the dressed-state scheme. Here we define *δx* = *x*′ − *x* as the deviation of *x*, in which *x* denotes the ideal value and *x*′ denotes the actual value. From Fig. 6(a), we learn that the fidelity slightly decreases with the increasing amplitude Ω_0_. It is clear that the increase of Ω_0_ causes the increase of the modified pulses’ amplitudes, and thus, to a certain extent, the Zeno limit condition will be spoiled. While simultaneously we can also see that, the longer *t*_*f*_ is, the higher the fidelity is. The reason can be deduced from Fig. 2 that when the operation time increases, the amplitudes of the modified pulses will decrease, and thus the Zeno limit condition will be met more strictly. Taking one with another, however, the fidelity always keeps near *F* = 1, and hence the dressed-state scheme have the extremely high robustness against the variations in *t*_*f*_ and Ω_0_. Analogously, the entanglement generation has a similar situation. Dissimilarly, however, the condition sin *θ*(*t*_*f*_) = cos *θ*(*t*_*f*_) (i.e., *θ*(*t*_*f*_) = *π*/4) in Eq. (11) is very critical for the maximum entanglement generation. Therefore, we are supposed to consider the effect of the variations in *θ*(*t*_*f*_) on the fidelity. We plot the fidelity versus the variations in *t*_*f*_ and *θ*(*t*_*f*_) in Fig. 6(b) for the entanglement generation based on the dressed-state scheme. To all appearances, in Fig. 6(b), the effect of the variations in *θ*(*t*_*f*_) on the fidelity is far greater than that of the variations in *t*_*f*_. But even so, the fidelity can keep quite high even when |*δθ*(*t*_*f*_)/*θ*(*t*_*f*_)| = 0.1. To sum up, the dressed-state scheme are robust against variations in the parameters of the pulse sequences.

**Figure 7:**
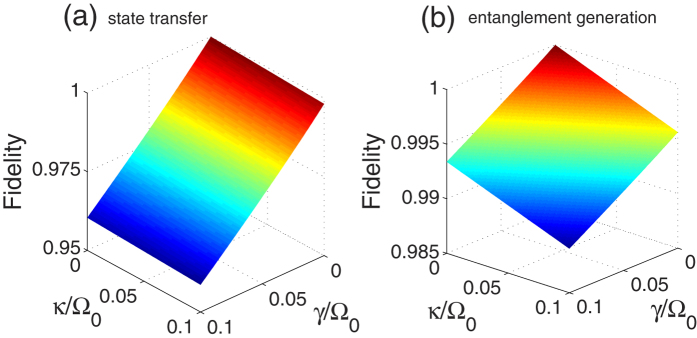
The fidelity as a function of *γ*/Ω_0_ and *κ*/Ω_0_.The parameters used here are the same as in Fig. 5.

Next, we take the decoherence induced by the atomic spontaneous emissions and the photon leakage from the cavity into account. Then the whole system is dominated by the master equation


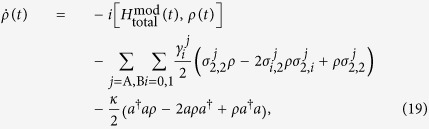


where 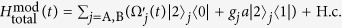; 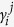 is the spontaneous emission rate of *j*th atom from the excited state |2〉_*j*_ to the ground state |*i*〉_*j*_; *κ* denotes the photon leakage rate from the cavity; 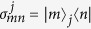. For simplicity, we assume 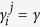. By means of the master equation, we plot the fidelities for the state transfer and the entanglement generation versus *κ*/Ω_0_ and *γ*/Ω_0_ in Fig. 7. Firstly, in Fig. 7, we can see that the fidelities for the state transfer and the entanglement generation are over 0.95 and 0.985, respectively, even when *κ* = *γ* = 0.1 Ω_0_. Without a doubt, the dressed-state scheme for the state transfer or the entanglement generation is robust against the decoherence induced by the atomic spontaneous emissions and the photon leakage from the cavity. In addition, as seen from the decrease of the fidelities with the increases of *κ* and *γ* in Fig. 7, we learn that the influence of the atomic spontaneous emissions on the fidelity is obviously greater than that of the photon leakages from the cavity. Even in Fig. 7(a), for the state transfer, the influence of the atomic spontaneous emissions on the fidelity almost plays a full role, but that of the photon leakages from the cavity is little. It follows that the dressed-state scheme we propose has the robustness against the photon leakages from the cavity more than the atomic spontaneous emissions.

**Figure 8:**
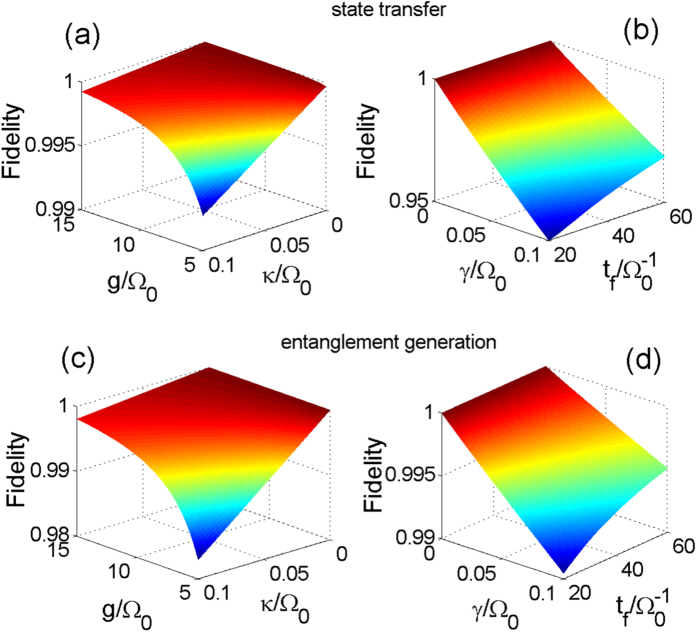
(**a**,**c**): The fidelities as functions of *g*/Ω_0_ and *κ*/Ω_0_ with *t*_*f*_ = 40/Ω_0_ and *γ* = 0; (**b**,**d**): the fidelities as functions of 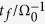 and *γ*/Ω_0_ with *g* = 10 Ω_0_ and *κ* = 0. Other parameters used here are the same as in Fig. 2.

We have chosen the Zeno limit condition 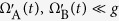 to restrain the population of the cavity-mode excited state |*ϕ*_3_〉. Besides, we have known that the increase of the operation time *t*_*f*_ leads to the decrease of |*μ*(*t*)|_max_ from Fig. 3(b), and thus leads to the decrease of |*ϕ*_*d*_〉 population sin^2^*μ*(*t*). Therefore, the influence of the photon leakages from the cavity and the atomic spontaneous emissions on the fidelity should be restrained by a bigger *g* and a longer *t*_*f*_, respectively. Based on this, we plot the fidelities as the functions with respect to *g* and *κ* in Fig. 8(a,c) for the state transfer and the entanglement generation, respectively. Clearly, when the photon leakages from the cavity exist, a bigger value of *g* can greatly depress the influence of the photon leakages from the cavity on the fidelities. In Fig. 8(b,d), we plot the fidelities as the functions with respect to *t*_*f*_ and *γ* for the state transfer and the entanglement generation, respectively. Also clearly, when the atomic spontaneous emissions exist, the influence of the atomic spontaneous emissions on the fidelities can be restrained by a longer *t*_*f*_. Nevertheless, for the experimental feasibility and the efficiency of the scheme, we pick an appropriate pair of values *g* = 10 Ω_0_ and *t*_*f*_ = 40/Ω_0_.

**Figure 9:**
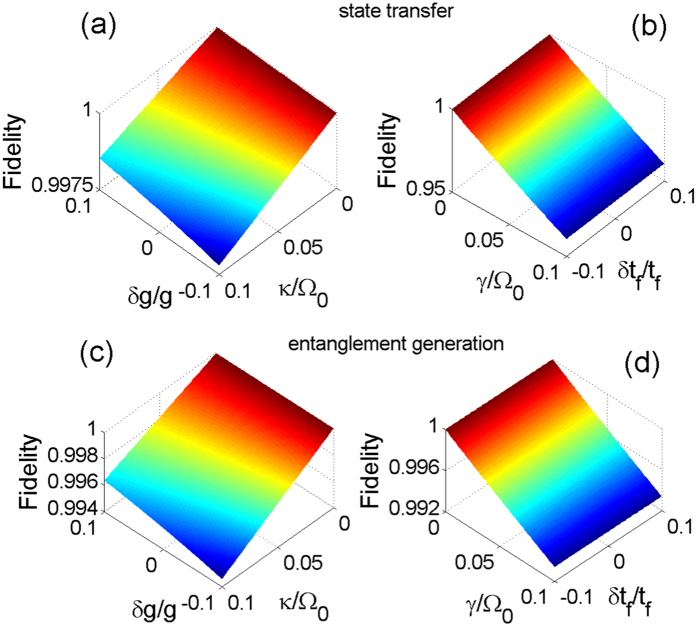
(**a**,**c**): The fidelities versus *κ*/Ω_0_ and the variations in *g* with *γ* = 0; (**b**,**d**): the fidelities versus *γ*/Ω_0_ and the variations in *t*_*f*_ with *κ* = 0. The parameters used here are the same as in Fig. 5.

Experimentally, it is too hard to have the theoretically predicted values of the parameters. Therefore, in Fig. 9, we give the numerical simulations to discuss the joint effects of the photon leakages from the cavity and the variations in the atom-cavity coupling strength *g* = 10 Ω_0_ and those of the atomic spontaneous emissions and the variations in the operation time *t*_*f*_ = 40/Ω_0_ on the fidelities. Figure 9(a,c) also indicate that when the photon leakages from the cavity exist, a bigger value of *g* can greatly depress the influence of the photon leakages from the cavity on the fidelities. Although either *κ* or the variations in *g* can not be controlled in experiment, the fidelities are over 0.99 even under the terrible condition {*δg*/*g* = −0.1, *κ* = 0.1 Ω_0_}. Figure 9(b,d) show that the fidelities are almost not affected by the variations in *t*_*f*_ = 40/Ω_0_ whenever the atomic spontaneous emissions exist or not. In other words, the dressed-state scheme are extremely robust against the variations in the chosen operation time. In addition, once the operation time is determined, the effects of the atomic spontaneous emissions on the fidelities are independent on the variations in the operation time.

## Conclusion

In conclusion, we have developed the dressed-state method to speed up the adiabatic quantum state transfer and entanglement generation between two Λ-type atoms trapped in an optical cavity. There is no a direct coupling of the target state and the initial state appearing in the Hamiltonian. The pulses are modified with the high experimental feasibility. During the whole evolution, the adiabatic condition is not necessary to be met, and thus even within a very short operation time, the state transfer and the entanglement generation can be achieved with quite high fidelities. The introductions of the Zeno limit condition and the auxiliary function *G*(*t*) restrain the populations of all of the excited states and hence the scheme is robust against the decoherence induced by the atomic spontaneous emissions and the photon leakage from the cavity. Besides, the results of the numerical simulations show that the dressed-state scheme is robust against the variations in the chosen parameters.

Based on ref. 46, by using cesium atoms and a set of cavity-QED predicted parameters (*g, κ, γ*)/2*π* = (750, 3.3, 2.62) MHz, we can achieve the two-atom quantum state transfer and maximum entanglement generation with the fidelities *F* = 0.985 and *F* = 0.996, respectively. Therefore, it allows us to construct an atomic system for the quantum state transfer and the entanglement generation in the presence of decoherence. In a word, by using dressed states, we have implemented the fast, feasible and robust two-atom adiabatic quantum state transfer and entanglement generation. In the further work, it could be interesting to apply the dressed-state method to more complex systems for preparing more complex entanglement and constructing quantum gates.

## Additional Information

**How to cite this article**: Wu, J.-L. *et al*. Fast adiabatic quantum state transfer and entanglement generation between two atoms via dressed states. *Sci. Rep.* **7**, 46255; doi: 10.1038/srep46255 (2017).

**Publisher's note:** Springer Nature remains neutral with regard to jurisdictional claims in published maps and institutional affiliations.
